# Stress and Resilience Development in German Dental Students

**DOI:** 10.1111/eje.70107

**Published:** 2026-02-11

**Authors:** Leonie X. Lin, Christine Keul, Martin R. Fischer, Jan Kiesewetter

**Affiliations:** ^1^ LMU Munich Munich Germany; ^2^ Department of Prosthodontics LMU Munich Munich Germany; ^3^ Institute of Medical Education (DAM) LMU University Hospital, LMU Munich Munich Germany

**Keywords:** dental education, dental schools, dental students, mental health, psychological resilience

## Abstract

**Introduction:**

The study evaluated the effectiveness of a resilience training for dental students as a preventive measure for mental health and stress management, with consideration for its long‐term integration into dental education.

**Methods:**

A 2‐day online resilience training was tailored to address specific stressors experienced by dental students in Munich, Germany. Data were collected via online questionnaires from both participants (pre and posttraining) and nonparticipant controls over a 6‐month period. Outcomes included resilience development, health behaviour, perceived stress and life satisfaction, utilising an online questionnaire. Statistical analysis (*t*‐tests and mixed ANOVA) was performed using IBM SPSS Statistics.

**Results:**

Training participants showed significantly increased resilience F(1.54) = 4.93, *p* = 0.031, η^2^ = 0.084 (medium effect), reduced subjective stress and higher overall satisfaction. Qualitative feedback indicated enhanced social support, though the effect on physical health behaviour remained inconclusive. Differences in resilience and satisfaction were observed between preclinical and clinical students, and between sexes.

**Discussion:**

This resilience training demonstrated preventive benefits for dental students' mental health. Limitations include selection bias, self‐reported measures, lack of long‐term data and confounding societal influences. Integrating such training into dental curricula may be a valuable strategy with potential for broader application in other institutions as well.

## 1 Introduction

Resilience, the ability to maintain health under stress or recover from challenges, is a dynamic and trainable process linked to mental well‐being [1, 2]. Healthcare professionals face high stress levels, starting in education due to time pressure, patient communication demands and ethical responsibilities [3, 4, 5, 6].

Dental students (*n* = 15 492) represent only 15% of medical students in Germany, yet receive less attention in mental health research despite facing similar stressors [7, 8, 9]. However, the existing body of research suggests that dental students and dentists in particular are susceptible to stress during their studies and professional lives [10, 11]. This susceptibility is attributed to the specific demands of the dental profession and personal predispositions of individuals who choose to become dentists [10, 12]. A recent study in Germany revealed that dental students show an increased risk of burnout based on emotional exhaustion and reduced personal performance, advocating a demand of specific interventions [13]. Recent studies further demonstrate that resilience and grit are important protective factors against burnout and are positively associated with well‐being in dental students [14]. Based on these considerations, the goal of this study is to develop, test and evaluate a suitable preventive measure in the form of an interventional resilience training specifically designed for dental students.

Following a survey at the Dental Clinic of Ludwig‐Maximilians‐University (LMU) Munich in spring 2020 that identified significant stressors in dental education (unpublished), this study addresses these stressors through a tailored intervention. In collaboration with the Institute of Medical Education (DAM) at LMU University Hospital, the ‘Resilience Training for Medical Students, Physicians, and Healthcare Professionals’ [15, 16] has been adapted and conducted with dental students in various semesters. Steps have been initiated to officially integrate the resilience training into the curriculum of dentistry at LMU, in alignment with the New National Licensing Regulations for Dentists (‘Zahnärztliche Approbationsordnung’ (ZApprO) [17]). This aims to instill comprehensive, mentally constructive health behaviour in students, ensuring the development of sustainably healthy dentists in Germany.

The objective of this work was to research and develop a specific resilience training programme for dental students as a potential health preventive intervention. Given that the research period aligns with the global COVID‐19 pandemic, various changes in individual and societal life since early 2020 can be summed up as additional stressors for everyone [18].

### 1.1 Hypotheses

The following hypotheses were raised in this study:

1. Dental students show increased resilience after completing the resilience training within dental school.
2. The training improves the (a) subjective health and (b) stress experiences as well as (c) life and work/study satisfaction of participants.
3. Sex and academic year are associated with measured resilience, subjective distress and overall life satisfaction.

## 2 Methods

Ethical approval was obtained from LMU's ethics board (18–535) and performed in accordance with the ethical standards of the institutional research committee and with the 1964 Helsinki declaration. All participants signed a consent form in which they agreed to be part of the study.

### 2.1 Study Design

This research involved a longitudinal study spanning four semesters, equating to 21 months, as well as a cross‐sectional study of all primary data.

(a) Longitudinal Study (Two‐Group Pre‐Post Design): For the intervention group, data were collected before and after participating in the resilience training, while the control group (students not partaking in training) was surveyed at the beginning and end of each semester. This design aimed to assess the effectiveness of the resilience training on the well‐being, stress levels and resilience development of dental students. G‐Power analysis suggested a minimum of 30 participants for statistically meaningful results using paired *t*‐tests and mixed ANOVA. Due to incomplete questionnaire data, the final sample size was set at 28 participants in both the intervention and control groups. Despite the reduced sample size, analyses were conducted based on anticipated large effect sizes.

(b) Cross‐Sectional Study: The questionnaire used generated cross‐sectional data for all participants at each measurement point, providing a comprehensive overview of the entire sample. To eliminate duplicates, only primary data from paired datasets were included in the analysis for this part of the study. The final sample consisted of *n* = 149 primary data questionnaires.

### 2.2 Timeline and Target Group

The initial resilience training sessions commenced in the summer semester of 2021. The study continued through the subsequent semesters up to and including the winter semester of 2022/2023. T1 refers to the pre‐intervention, or 1st assessment (by questionnaire) and was conducted in the beginning of each academic semester, T2 refers to the post‐intervention, or 2nd assessment at the end of each semester. The intervention (training) was held in the middle of each semester. The study timeline is summarised as illustrated in Figure 1.

**FIGURE 1:**
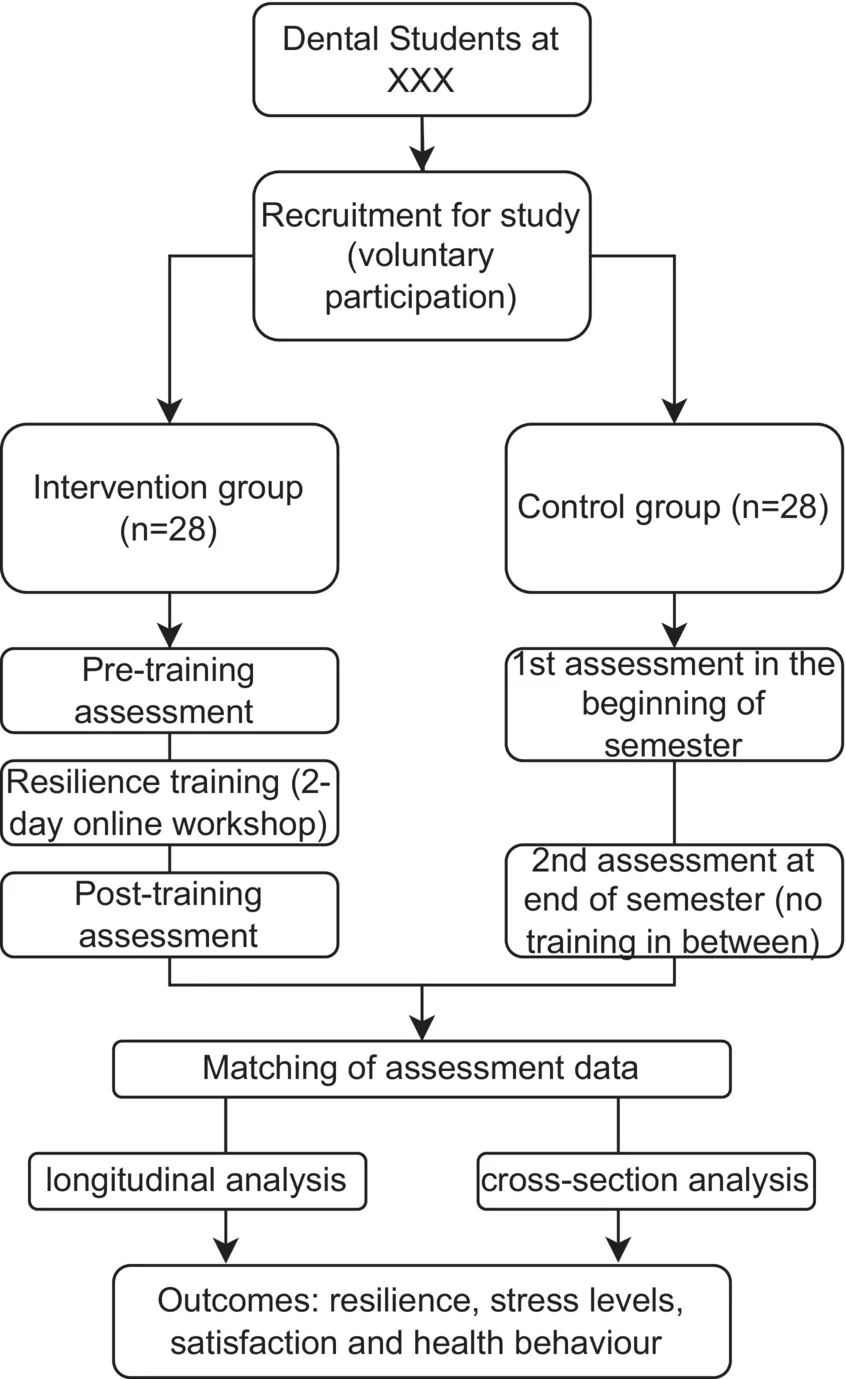
Flow chart of study design (own creation).

The target group for both the surveys and the resilience training comprised exclusively dental students at LMU Dental Clinic (total amount of students at the time of study *N* = 671). Sampling was conducted on a voluntary basis, with students self‐selecting into either group based on participation in the resilience training. Completion of the questionnaires was voluntary and anonymous. Response rate was 22.2%.

### 2.3 Data Collection Instruments

The software ‘EvaSys’ was used for data collection. The research employed a battery of standardised questionnaires to address the research questions, which took approximately 10 min to complete:

1. Questionnaire on Stress Management and Quality of Life in Medical Studies (STQL‐S) [19]: The STQL‐S is a 71‐item German questionnaire developed by the Center for Psychosomatic Medicine at LMU, specifically tailored to medical and dental students. It has been used in similar university settings across Germany and covers multiple dimensions, including subjective academic and personal stress, life satisfaction, study‐related anxieties, motivations, lifestyle habits and coping behaviour. Topics include academic satisfaction, private life, recreational and stress management strategies, health‐related behaviours (e.g., sleep, substance use, physical activity) and contextual factors such as study location and career plans. Data are collected through a mix of closed‐ended formats, Likert scales, dichotomous and multiple‐choice items. Since no total score or standardised classification exists, interpretation relies on comparison across subscales and between participant groups. While the STQL‐S has been applied in various empirical studies in Germany, formal reliability and validity data have not yet been published.
2. 10‐Item Version (German) of the Connor‐Davidson Resilience Scale (CD‐RISC‐10) [20]: The CD‐RISC‐10, a widely used instrument to assess resilience, consists of 10 items. Its German version shows good reliability and validity [21]. Responses were scored on a 0–4 Likert scale, resulting in a score range of 0–40. The CD‐RISC‐10 measures resilience across five domains, including personal competence and endurance, tolerance of negative effects, control, trust in one's instincts and spirituality. High scores indicate higher resilience. There are no standardised cut‐off scores for classification, so any proposed thresholds should be interpreted cautiously and within the context of the control groups.

For additional insights, the researchers considered other questionnaires, such as the Perceived Medical School Stress Instrument (PMSS‐D) [22], Dental Environmental Stress (DES) [23] and Academic Resilience Scale (ARS‐30) [24] but ultimately excluded them due to various factors, including cost and duplication of content.

### 2.4 Resilience Training

Content of the resilience training: The adapted resilience training for dental students comprises five modules, each with a duration of approximately 2 h, allowing for short breaks within a training day or distribution over an extended period. The training modules are as follows:

1. Time and energy management: Focusing on participants' self‐awareness and time management, exploring the connection between time allocation and energy management. Emphasis is placed on a comprehensive view of health, covering physical, emotional, cognitive and spiritual aspects [15]. Additionally, topics such as salutogenesis [25] and sense of coherence [26] in the context of dental education in Germany are discussed.
2. Resilience and mindfulness: Addressing the link between resilience and mindfulness, introducing simple mindfulness strategies and exploring their integration into daily life. The five resilience factors—adaptability, self‐control, perseverance, optimism and self‐efficacy—are discussed in the context of dental studies [15]. The module also includes the development of stress reduction strategies specific to dental education.
3. Performance‐related emotions: Examining performance‐related emotions, attribution and the impact of thoughts on feelings. Participants are encouraged to engage in open discussions about their perspectives, behaviours and thought patterns in the context of their studies [15]. Social challenges and stressors related to competition and hierarchies in dental clinics are addressed. Furthermore, participants explore their inner motivators and belief systems, and these concepts are adapted to the context of dental education.
4. Coping with emotions: Focusing on strategies to manage stress and setbacks, as well as strong, negative emotions in the academic setting. This module introduces various mental strategies and the transactional stress and coping model by Lazarus and Folkman [27]. The ‘Emotional Speed Dating’ exercise [15] is employed to identify and develop coping mechanisms for challenging situations that dental students commonly encounter.
5. Work–Life Harmony: The final module emphasises the integration of the acquired knowledge into participants' lives. It involves revisiting individual time and energy management, goal setting and motivation. This module provides a platform for reflection and sharing of learning experiences among participants.

Recruitment and advertising: The resilience training programme was promoted through digital posters, emails and word of mouth to dental students at LMU Dental Clinic. Promotion occurred at the beginning of each semester, with digital and manual posters being shared in all semester groups. Email invitations were sent by the dental programme curricular office to all students. Personal communication and word‐of‐mouth referrals among dental students further contributed to recruitment. Presentations were made to newly enrolled dental students during orientation events.

Training implementation: The training was led by health care professionals (dentists and psychologists) who had received specialised training based on a validated resilience training manual [15, 16]. The training was designed to accommodate 8–10 participants per session, offering flexibility in training delivery format. Offered as an online workshop due to COVID‐19 restrictions, participants found this arrangement practical and efficient to take part in.

### 2.5 Data Analysis

The quantitative data analysis of the survey results was conducted using IBM SPSS Statistics, Version 29, applying *t*‐tests and mixed ANOVA. The following steps were taken to process and clean the data:

1. Data integration: Data from seven survey time points, each with identical questionnaires over 21 months, were merged into a common database.
2. Data cleansing: Standardisation of capitalisation in individual identifiers (passwords) and correction of any minor typographical errors made by respondents to facilitate data pairing. Identifying related datasets was based on questionnaire items like password, sex, age, family history in dentistry and age at the time of study decision.
3. Demographic data inclusion: Demographic and personal descriptor items (sex, age, high school grade, study progress, pre‐clinical exam score, regular study time adherence) were added to each data pair to ensure consistency
4. Exclusion of invalid cases: Incomplete or illogical datasets were excluded.
5. Subdivision: The overall database was divided into smaller databases to allow various analyses.

#### 2.5.1 Descriptive Statistics

All primary data as part of the cross‐sectional study were initially analysed descriptively. For categorical variables, absolute and percentage frequencies were calculated. For ordinal or metric variables, means (M) with standard deviations (SD) were computed. Likert scale items, with values ranging from 1 (‘strongly agree’) to 4 (‘strongly disagree’), were reported as mean values (M ± SD) in the descriptive statistics.

#### 2.5.2 Conclusive Statistics

The normal distribution of samples from various study groups was assessed. Assuming a normal distribution, parametric tests such as paired *t*‐tests and mixed ANOVA were used. Nonparametric tests were not needed based on the sample results. The significance level for all statistical analyses was set at *α* = 0.05. No adjustment of the significance level for multiple tests was made due to the exploratory nature of the study.

To determine the practical significance of the effect, effect sizes were calculated for statistically significant results. Effect size interpretation followed Cohen [28], where *d* = 0.2 represents a small effect, *d* = 0.5 a medium effect and *d* ≥ 0.8 a large effect for *t‐*tests. For mixed ANOVA, effect size interpretation was based on the calculation of partial eta squared (*η* ^2^): 0.01 denotes a small effect, 0.06 a medium effect and ≥ 0.14 a large effect [28].

#### 2.5.3 Analysis of Qualitative Data

The free text entries were qualitatively analysed through categorisation [29]. The comments were initially categorised, tabulated, sorted by content and annotated. Categories were derived by grouping comments, and key statements relevant to the hypotheses were extracted and implemented in the following sessions.

## 3 Results

Of the total primary dataset (*n* = 149), 50% of participants were in the pre‐clinical phase (semesters 1–5) and 50% in the clinical phase (semester 6 onward). The distribution across semesters 1–10 was balanced in both the intervention and control groups. Overall, 85% of participants identified as female and 15% as male. Within the intervention group, 86% were female; in the control group, 82% were female.

(1) Dental students show increased resilience after completing the resilience training within dental school.

Based on the results obtained from the Mixed Analysis of Variance (ANOVA) for the CD‐RISC total score (total scores of the 10 items of the CD‐RISC), it has been confirmed that the measured resilience values of the intervention group showed a positive development over the study period between T1 and T2 (mean total score intervention group T1 = 22.9, T2 = 25.6), whereas the corresponding values of the control group showed a slight decline (mean total score control group T1 = 25.4; T2 = 24.3) (indicated in bold, Table 1). The interaction between time and study groups is statistically significant with a moderate effect size (*p* = 0.031, partial η^2^ = 0.084).

**TABLE 1:** CD‐RISC‐10 with tests on normal distribution (own creation).

CD‐RISC‐10 sum scale	Sample group	*N*	MW	SD	Shapiro–Wilk		
					Statistics	df	Significance
*CD‐RISC‐10 T1*	Intervention group	28	**22.89**	5.801	0.970	28	0.573
	Control group	28	**25.43**	7.270	0.968	28	0.519
	total	56	24.16	6.641			
*CD‐RISC‐10 T2*	Intervention group	28	**25.57**	8.297	0.954	28	0.255
	Control group	28	**24.39**	7.866	0.966	28	0.474
	total	56	24.98	8.033			

(2) The training improves the (a) subjective health and (b) stress experiences as well as (c) life and work/study satisfaction of participants.

Qualitative changes in health behaviour were observed. Due to the small sample size and as only qualitative patterns of behaviour were assessed with response options of yes, no, or occasionally, conclusive statistical tests for testing Hypothesis (2a) were not performed.

In the intervention group, the number of respondents who reported smoking and/or drinking alcohol decreased. At the same time, the number of respondents who reported engaging in regular physical activity and/or sports increased. Meanwhile, there was also an increase in those reporting the use of drugs and/or medications.

In the control group, there was no change in the number of smokers; however, respondents who reported engaging in regular physical activity, drinking alcohol and using drugs and/or medications decreased. The number of respondents in the control group who engaged in regular sports activities, on the other hand, increased.

(2b) Subjectively experienced stress decreased for participants at T2 after the resilience compared to T1 before the intervention. Meanwhile, the control group showed higher levels of stress at T2 end of semester compared to T1 beginning of semester (indicated in bold, Table 2). In the parametric testing using Mixed ANOVA, there was no statistically significant interaction effect between time and study groups (F(1.54) = 3.20, *p* = 0.079, partial η^2^ = 0.056).

**TABLE 2:** Stress scale (subscale from (STQL‐S) [19]) with tests on normal distribution (own creation).

	Sample group	*N*	MW	SD	Shapiro–Wilk		
					Statistics	df	Significance
*T1 stress scale*	Intervention group	28	**7.14**	2.563	0.958	28	0.316
	Control group	28	**8.29**	2.441	0.944	28	0.139
	Total	56	7.43	2.484			
*T2 stress scale*	Intervention group	28	**8.29**	2.522	0.931	28	0.064
	Control group	28	**7.50**	2.487	0.927	28	0.051
	Total	56	7.89	2.513			

*Note:* Descriptive statistics on stress scale (sub scale from (STQL‐S) [19]), Likert 1 to 15 (1 indicating highest, 15 indicating lowest levels of subjectively experienced stress).

(2c) The intervention group showed an increase in the measured overall satisfaction. Conversely, in the control group, there was a slight decrease (indicated in bold, Table 3). Through parametric Mixed ANOVA testing of the overall satisfaction indicators, a statistically significant interaction between time and study groups was found, assuming sphericity F(1.54) = 4.11, *p* = 0.048, hence *p* < 0.05, partial η^2^ = 0.071 (medium effect).

**TABLE 3:** Satisfaction scale (subscale from (STQL‐S) [19]) with tests on normal distribution (own creation).

	Sample group	*N*	MW	SD	Shapiro–Wilk		
					Statistics	df	Significance
*T1 Subskala Zufriedenheit*	Intervention group	28	**32.57**	7.416	0.940	28	0.110
	Control group	28	**30.68**	6.577	0.967	28	0.509
	Total	56	31.63	7.011			
*T2 Subskala Zufriedenheit*	Intervention group	28	**30.39**	8.015	0.903	28	0.013
	Control group	28	**31.04**	8.025	0.956	28	0.285
	Total	56	30.71	7.954			

*Note:* Descriptive statistics on life and work/study satisfaction scale (sub scale from (STQL‐S) [19]), Likert 1 to 65 (1 indicating highest, 65 indicating lowest levels of satisfaction).

(3) Sex and academic year are associated with measured resilience, subjective distress and overall life satisfaction.

The measured resilience total scores for male respondents (*n* = 22) were, on average, higher than those for female respondents (*n* = 125) (27.41 ± 6.745 vs. 24.50 ± 6.348). Additionally, male respondents appeared to experience less stress than female respondents (7.50 ± 3.113 vs. 7.21 ± 2.315). Satisfaction scores were very similar, albeit slightly lower for male respondents compared to female respondents (31.64 ± 8.533 vs. 31.74 ± 6.714). All three differences showed no statistical significance in the independent samples *t*‐tests (*p* > 0.05).

Students in the clinical study phase (semester 6 onwards) (*n* = 73) appeared to be more resilient (25.30 ± 6.191 vs. 24.66 ± 6.758), experiencing higher stress levels (7.21 ± 2.409 vs. 7.34 ± 2.473), yet also expressing higher overall satisfaction (32.25 ± 6.936 vs. 31.17 ± 7.073) compared to students in the pre‐clinical study phase (semester 1 to 5) (*n* = 73). The differences showed no statistical significance concerning the measured values for resilience, stress and satisfaction (*p* > 0.05).

## 4 Discussion

This study examined the effects of a tailored resilience training on dental students' psychological well‐being. Results showed a statistically significant increase in resilience scores and overall satisfaction, along with a reduction in subjectively perceived stress among participants. These findings suggest that resilience training can serve as an effective preventive measure for supporting mental health in dental education.

Using the test battery from the Questionnaire on Stress Management and Quality of Life in Medical Studies (STQL‐S) and the German version of the 10‐item Connor‐Davidson Resilience Scale (CD‐RISC), specific effects of the resilience training on health behaviour, stress experience and measurable resilience in dental students at LMU were found. Dental students at LMU scored lower in resilience compared to peers in other disciplines, consistent with existing data on elevated stress in dental education, suggesting resilience‐enhancing [30, 31, 32, 33, 34]. Other studies show that dental students, both internationally and in Germany, are particularly susceptible to stress compared to peers in general medicine and health sciences [4, 10]. In addition to the psychological outcomes, qualitative trends in health behaviour—such as a reduction in smoking and an increase in physical activity—were observed in the intervention group. Although these findings are descriptive and not statistically tested, they may indicate early behavioural changes accompanying resilience enhancement.

This underlines the importance of integrating effective health preventive measures, such as resilience training, into the dental curriculum. The transition to the new ZApprO [17] provides an opportunity for the initial implementation of such intervention programmes, introducing interdisciplinary offerings and new elective courses for all dental students nationwide.

The adapted resilience training comprehensively addresses the understanding of health, delving into the concepts of mental health, stress and resilience. Across training sessions, participants showed particular interest in distinct strategies for a healthier lifestyle. The World Health Organization's (WHO) three‐dimensional health definition [35] became a focal point, allowing participants to discuss individual connections between their holistic health status and the specifics of dental education. Participants praised the open communication facilitated during the resilience training as unique, offering a rare space for individual reflection and interdisciplinary exchange in the dental students' daily lives.

Quantitative data analysis revealed that all participants, regardless of training, perceived themselves as healthier, based on the WHO's health definition [35], at the follow‐up assessment compared to the primary assessment. However, advanced students in the clinical phase (semester 6 onward) reported lower health satisfaction, aligning with a parallel decline in overall life satisfaction over the study period. This shift may be attributed to increased awareness of health definitions and other interconnected stress and resilience components, potentially influenced by a truth and repetition effect/bias in repeated surveys [36].

Regarding mental health, the adapted resilience training emphasises concepts such as self‐efficacy, salutogenesis and sense of coherence, closely linked to life satisfaction and positive emotions [37]. Results showed a statistically significant increase in overall satisfaction and a decrease in perceived stress in the intervention group after resilience training compared to the nontrained control group. However, students in the clinical phase reported higher stress levels and lower overall satisfaction, mirroring the negative association between stress and satisfaction [38]. Although anxiety and suicidal thoughts showed slight nonsignificant improvement in the intervention group, the questionnaire does not adequately capture such psychological aspects due to its subjective, qualitative character.

While the resilience training incorporated strategies for physical health, no clear training‐attributed changes were observed in measured objective health behaviours (exercise, nutrition, substance use, sleep). The study emphasises that, as a primary preventive intervention [39], the resilience training primarily aims to reduce illness occurrence in a generally healthy student population. Given the emphasis on cognitive‐mental behavioural changes, it is not surprising that the training does not serve as an effective preventive measure for physical health.

Notably, other studies on students' health, especially since the COVID‐19 pandemic, which coincides with the timeframe of this study, have observed similar slight changes in health behaviour [40]. Whether and to what extent the described behavioural changes in the surveyed dental students are related to the offered resilience training, or rather to general societal development during times of a global pandemic, remains questionable.

Regarding social well‐being, dental students reported satisfaction with the quality of their social circles and private lives. Notably, the intervention group demonstrated an improvement in private life satisfaction compared to the nontrained group. The training's focus on social integration and support likely contributed to this positive trend. Qualitative feedback also highlighted the value of social exchange, reducing the feeling of isolation in stressful situations. This is valuable because there exists a positive correlation between psychosocial competencies and increased stress coping and well‐being in academic settings [41].

The presented results demonstrate that the conducted resilience training exhibited a statistically significant positive effect on the resilience development of the participants, as anticipated. Whether this represents a sustainable long‐term development remains uncertain at the current time due to a lack of long‐term data.

In the longitudinal study design, the intervention group showed markedly increased scores in the resilience sum scale after completing the training, compared to the nontrained control group. In comparison to the resilience improvements in the intervention group, there were only slight and not unidirectional differences in the resilience values of the nontrained control group. These minor fluctuations seem normal over the 6‐month investigation period, considering resilience as a variable or dynamic process.

In light of other studies on student health since the COVID‐19 pandemic, which observed a downward spiral in mental health indicators, increased loneliness and (social) stress [42], the improvements in resilience, experienced stress and overall satisfaction shown through the resilience training become even more valuable.

Remarkably, across all study groups, the measured overall resilience according to the CD‐RISC sum scale remains lower than in surveyed students and peers of other disciplines [21]. Whether this results from methodological limitations, sampling bias, or genuinely lower resilience levels of the study group remains unanswered. Although the adapted resilience training has been confirmed in its effectiveness for participating dental students in Munich, Germany, it is important to emphasise that this specific resilience training does not exclude the possibility of developing other, potentially more efficient preventive measures for the context of dentistry (and dental education).

The greatest limitation of the data collection is a presumed selection bias. Both participation in resilience training and surveys were voluntary and anonymous. It can be assumed that students with a prior interest in the topic were more likely to participate in both the training and the surveys. Although uniform invitations were sent by email to all dental students at the LMU Dental Clinic, it cannot be traced to what extent these invitations were received by all dental students.

Further limitations include the subjectivity with which all participants answered the questionnaire. All qualitative answers, such as regarding health behaviour, distress, resilience and satisfaction, are subject to the experiences and self‐perception of the participants. However, especially regarding mental health questions, such as about distress and resilience, this subjectivity is essential for measurement.

## 5 Conclusion

This study demonstrates that a targeted resilience training programme significantly improves psychological resilience, reduces stress and increases satisfaction among dental students. These effects are particularly relevant given the high stress levels documented in dental education [10]. The measurable outcomes directly reflect the focus of the training and support the integration of structured mental health interventions into the dental curriculum.

Differences between preclinical and clinical students suggest the value of stage‐specific adaptations of the programme [13]. Embedding resilience training within the new national licensing framework (ZApprO) offers a timely opportunity to address student well‐being on a broader scale [17].

Despite the study's limitations—self‐selection bias and reliance on self‐reported data—the consistency between quantitative results and student feedback supports the intervention's effectiveness. The programme's focus on open discussion and applied coping strategies may have been key to its impact.

In summary, resilience training represents a scalable, evidence‐based approach to improving mental well‐being in dental education. These results provide a foundation for curricular innovation and long‐term support strategies tailored to the needs of dental students.

## Funding

The authors have nothing to report.

## Conflicts of Interest

The authors declare no conflicts of interest.

## Data Availability

The data that support the findings of this study are available on request from the corresponding author. The data are not publicly available due to privacy or ethical restrictions.
